# Breast Cancer Classification Using Feature Selection via Improved Simulated Annealing and SVM Classifier

**DOI:** 10.3390/diagnostics16040637

**Published:** 2026-02-23

**Authors:** Maedeh Kiani Sarkaleh, Hossein Azgomi, Azadeh Kiani-Sarkaleh

**Affiliations:** 1 Department of Computer Engineering, Ra.C., Islamic Azad University, Rasht 4147654949, Iran; m.kiani8312@iau.ac.ir; 2 Department of Electrical Engineering, Ra.C., Islamic Azad University, Rasht 4147654949, Iran; azadeh.kiani@iau.ac.ir

**Keywords:** breast cancer, image processing, improved simulated annealing, feature selection, SVM classifier

## Abstract

**Background:** Breast cancer is among the most common cancers in women, and early diagnosis is critical for better treatment outcomes and reduced mortality. Efficient computer-aided diagnostic (CAD) systems play a crucial role in enhancing diagnostic accuracy and facilitating timely clinical decisions. **Methods:** This study proposes an automated CAD system for detecting cancerous tumors in mammograms, consisting of four stages: preprocessing, feature extraction, feature selection, and classification. In preprocessing, the region of interest (ROI) is extracted, followed by noise suppression and contrast enhancement to improve image quality. Shape, histogram, and tissue-related features are then computed from each ROI. An Improved Simulated Annealing (ISA) algorithm is employed to adaptively select the most informative features through a flexible process and composite fitness function, effectively reducing dimensionality while preserving high classification accuracy. Finally, classification is performed using a Support Vector Machine (SVM) to distinguish between malignant and benign masses. **Results:** Evaluation on the CBIS-DDSM and MIAS datasets showed the system achieved accuracies of 99.67% and 98%, sensitivities of 99.33% and 98%, and F1-scores of 99.66% and 97.9%, respectively. These results indicate notable improvements over traditional SA and full-feature approaches. **Conclusions:** The findings confirm the effectiveness of the ISA algorithm in selecting relevant features, thereby enhancing the performance of breast cancer detection.

## 1. Introduction

Despite significant advancements in early diagnosis and medical treatments, breast cancer remains the second leading cause of death among women worldwide and the primary cause among Black and Hispanic women. Data indicate that the breast cancer mortality rate declined by approximately 44% from 1989 to 2022, largely due to improved medical practices, more precise screening, and early detection [1]. Breast cancer is characterized by the uncontrolled proliferation of abnormal cells in breast tissue, leading to tumor formation. If left undiagnosed and untreated, these tumors can metastasize to other parts of the body, posing a serious threat to the patient’s health. The prevalence of breast cancer continues to rise globally, with the highest incidence observed in industrialized countries. This trend is influenced by various factors, including lifestyle, age, hormones, genetics, and dietary habits [2].

Early diagnosis of breast cancer enables the timely initiation of treatment, significantly increasing the chances of success. Even in regions with limited access to specialists, machine learning can support early detection [3]. Mammography remains a fundamental tool for breast cancer screening; however, interpreting masses in mammographic images presents a significant challenge. To address this, Computer-Aided Diagnosis (CAD) systems have been developed to enhance the detection and classification of breast masses. These systems serve as assistive tools for radiologists, helping identify anomalies such as tumors or microcalcifications in digital mammography.

This paper presents a system for detecting cancerous masses in mammographic images. The proposed approach identifies tumors by analyzing suspicious regions within the images. It is composed of four steps: preprocessing, feature extraction, feature selection, and classification. In the preprocessing step, after the Region of Interest (ROI) is extracted from the image, a median filter is applied to eliminate noise, and histogram equalization is used to improve contrast. Next, shape, histogram, and tissue features are extracted from the ROI. The feature selection step utilizes the Simulated Annealing (SA) algorithm. Finally, the system classifies the detected masses using the Support Vector Machine (SVM) classifier.

SA is a metaheuristic optimization algorithm introduced by Kirkpatrick et al. [4] for searching large and discrete solution spaces. The algorithm is inspired by the metallic annealing process, in which metals are heated to high temperatures and then gradually cooled. At elevated temperatures, atomic movement increases rapidly, while controlled cooling enables the formation of specific patterns in their positions. This study leverages this characteristic of SA for feature selection, ensuring the identification of the most relevant features. The gradual cooling mechanism in SA reduces the likelihood of selecting suboptimal solutions and facilitates finding an optimal solution. The fitness function evaluates the quality of the extracted features, with the optimal ones selected as the final output. The optimization process begins by setting an initial temperature, which is then progressively reduced. During the search, the algorithm iterates through potential solutions, refining them while occasionally accepting less optimal candidates with a defined probability to avoid falling into the trap of local optima.

This study presents a system based on mammographic image processing and feature optimization via the Improved Simulated Annealing (ISA) algorithm. The system consists of preprocessing, feature extraction, feature selection, and classification using an SVM, aiming to enhance detection accuracy by selecting the optimal features. Leveraging a composite cost function, the ISA algorithm effectively determines the number of selected features, eliminating the need for manual selection while extracting the most relevant characteristics from the initial feature set. This approach significantly enhances classification performance by effectively balancing dimensionality reduction and accuracy improvement. Experimental results validate its efficiency.

The contributions of this study are as follows:

- Development of a breast tumor detection system based on mammographic image analysis, utilizing the SA algorithm for feature selection and SVM for classification
- The application of a composite set of histograms, shape, and tissue features to enhance tumor detection accuracy
- Dimensionality reduction of the feature space through optimal feature selection via the ISA algorithm, improving classification accuracy while minimizing computational complexity

The paper is structured as follows. Section 2 reviews the relevant literature on breast cancer diagnosis. Section 3 outlines the proposed method, which consists of four steps: preprocessing, feature extraction, feature selection, and classification. Section 4 presents the results of implementing the proposed system and evaluates its performance. Finally, Section 5 provides a summary of the paper and offers recommendations for future work.

## 2. Related Works

Numerous CAD systems have been developed for the analysis of digital mammograms, aiming to improve radiologists’ performance in breast cancer detection [5]. These systems employ various feature selection algorithms to extract an optimal subset for breast tumor classification. This section reviews studies on the application of machine learning and metaheuristic optimization methods in breast cancer diagnosis.

The system proposed in [6] for breast cancer detection integrates a synthesized Convolutional Neural Network (CNN), an advanced optimization algorithm, and transfer learning. The research utilizes a metaheuristic Ant Colony Optimization (ACO) algorithm, enhanced by Opposition-Based Learning (OBL), to optimize hyperparameter selection in the CNN architecture. Experimental results on the MIAS and CBIS-DDSM datasets demonstrate accuracy rates of 99.15% and 98.63%, sensitivity of 97.86% and 98.76%, and specificity of 98.88% and 98.89%, respectively.

A deep learning-based approach was introduced in [7] for malignant breast tumor detection, employing the metaheuristic Grasshopper Optimization Algorithm (GOA) for optimal feature selection. Evaluation on the MIAS and DDSM datasets yielded 96% accuracy, 97% specificity, 75% sensitivity, 99% positive prediction value (PPV), and 45% negative prediction value (NPV).

The system described in [8] integrates deep learning models with optimization algorithms to enhance the detection of breast cancer tumors. It employs CNNs, specifically DenseNet-121 and VGG-16, for feature extraction, while bidirectional long short-term memory (BiLSTM) layers enhance temporal feature extraction. Parameter tuning is conducted using a modified Grey Wolf Optimization algorithm, and classification is performed using SVM and Random Forest (RF) classifiers. Performance evaluation, based on metrics such as accuracy, sensitivity, specificity, precision, and area under the curve (AUC), yielded results of 99.86%, 99.9%, 99.7%, 97.1%, and 1.0 on the MIAS dataset, and 99.4%, 99.03%, 99.2%, 97.4%, and 1.0 on the INbreast dataset, respectively.

A system has been proposed in [9] to enhance breast cancer detection accuracy by integrating the Quantum-inspired Binary Grey Wolf Optimizer and Radial Basis Function (RBF) with SVM. Evaluated on the MIAS dataset, this approach achieved 99.25% accuracy, 98.96% sensitivity, and 100% specificity.

In [10], a system for detecting mammographic anomalies and classifying breast tumors as benign or malignant is introduced. It utilizes the Curvelet transform and fractal analysis for feature extraction, alongside a genetic algorithm-based multi-objective optimization approach for optimal feature selection and artificial neural network training. Evaluation on the MIAS dataset demonstrated an accuracy of 98.2%, specificity of 100%, sensitivity of 96%, PPV of 100%, NPV of 96.2%, and an AUC of 0.98.

A feature selection framework based on adaptive teaching-learning strategies is proposed in [11] for breast cancer detection from mammographic images. This method employs Weighted Adaptive Binary Teaching-Learning-Based Optimization (WA-BTLBO) to select the most relevant features, with classification accuracy serving as the fitness function. The selected features are evaluated using the XGBoost classifier, and the performance is subsequently compared with that of other well-established classifiers, including SVM, RF, KNN, and ANN. Performance assessment on the MIAS dataset showed that XGBoost delivered the highest accuracy at 97.8%, while SVM, RF, KNN, and ANN achieved 93.8%, 94.8%, 92.7%, and 93.9%, respectively.

In [12], a segmentation and classification framework for breast cancer identification, termed Optimized Graph Convolutional Recurrent Neural Network-based Segmentation for Breast Cancer Recognition and Classification (OGCRNN-SBCRC), is presented. Wiener filtering and log transform are applied in the preprocessing stage to reduce noise and enhance image contrast. The segmentation process is carried out using the ++UNet architecture, with parameter optimization handled by the RMSProp algorithm. Feature extraction is performed by the ConvNeXtTiny network, while classification is conducted using the Graph Convolutional Recurrent Neural Network (GCRNN). Additionally, hyperparameters are fine-tuned using the Aquila Optimizer (AO). Evaluation on the CBIS-DDSM dataset indicates an accuracy of 99.65%.

In [3], a three-stage framework for breast cancer detection and classification is introduced, consisting of preprocessing, hyperparameter optimization, and model training. During preprocessing, non-breast regions are eliminated, and noise reduction is achieved through median filtering and Contrast Limited Adaptive Histogram Equalization (CLAHE) to enhance image contrast. Hyperparameters are optimized using the Hunger Games Search (HGS) and GWO algorithms, while feature extraction is performed via DenseNet-121 and EfficientNet-B5 architectures. Finally, classification is conducted using SVM. Performance evaluation on the INbreast dataset yielded an accuracy of 99.9%, sensitivity of 99.9%, specificity of 99.8%, PPV of 99.1%, and an AUC of 1.0.

A review of the literature indicates that advancements in breast cancer detection systems have primarily been driven by optimizing model parameters and selecting effective features. However, many existing methods either suffer from excessive computational complexity or fail to achieve optimal feature selection. The Simulated Annealing (SA) algorithm offers an effective and computationally efficient approach for identifying a subset of key features, reducing data dimensionality, and enhancing classification accuracy. Accordingly, this study focuses on leveraging the capabilities of the SA algorithm and SVM classifier to achieve optimal feature selection and precise detection of malignant tumors in mammographic images.

## 3. Proposed Method

**Figure 1:**
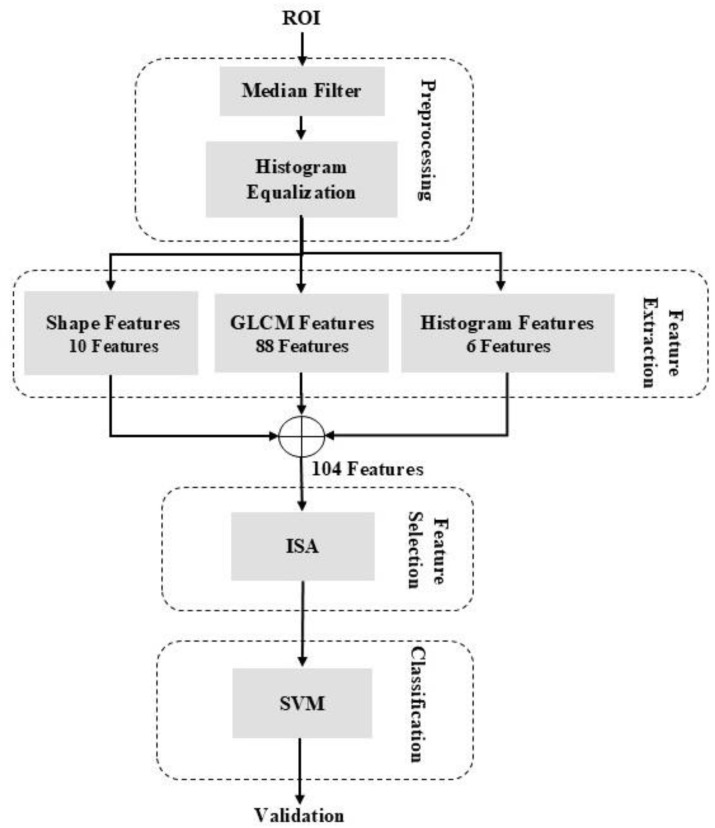
The flowchart of the proposed method.

This paper presents a CAD system for identifying cancerous tumors in mammographic images. As illustrated in Figure 1, the system consists of four primary stages: preprocessing, feature extraction, feature selection, and classification.

In the preprocessing stage, the ROI is first isolated from the image. Generally, ROI extraction is performed using either clustering or cutting techniques. The clustering approach uses image processing techniques to automatically identify suspicious regions, while the cutting method leverages spatial data within the dataset to accurately segment the tumor area. Given the availability of spatial data in the dataset, this study employed the cutting method for ROI extraction. Following ROI isolation, image quality is enhanced through median filtering for noise reduction and histogram expansion for contrast improvement. During feature extraction, shape, histogram, and tissue-related features within the ROI are extracted. Feature dimensionality is then reduced, and the most relevant features are selected using the SA algorithm. Finally, classification is performed using SVM to distinguish cancerous masses from non-cancerous ones.

### 3.1. Preprocessing

In the preprocessing stage, median filtering is initially applied to enhance the quality of mammographic images and improve detection accuracy. This nonlinear filter effectively removes random noise by replacing each pixel’s intensity value with the median of its neighboring values. A key advantage of median filtering over other noise-reduction techniques is its ability to minimize image distortion. Specifically, it preserves critical details and sensitive edges, ensuring that delicate structures such as microcalcifications, which are essential for early breast cancer detection, remain intact and non-blurred. In this study, a 3 × 3 square kernel was utilized for median filtering. Following noise removal, histogram equalization is employed to enhance image contrast and expand intensity levels. By redistributing intensity values, this technique enhances the visibility of both dark and bright regions, revealing finer details and significantly improving the accuracy of subsequent processing and classification steps [13].

### 3.2. Feature Extraction

Feature extraction, a critical stage in medical image detection systems, aims to generate a precise representation of image characteristics. The extracted features must exhibit strong discriminative power to ensure maximal intra-class similarity and inter-class separability, thereby enhancing classifier performance. Benign and malignant tumors in mammograms exhibit distinct geometric and textural properties. Benign tumors typically have well-defined, regular shapes (circular or elliptical) with smooth, clearly delineated margins, whereas malignant tumors present irregular shapes with indistinct, poorly defined edges. Additionally, these two tumor types differ in tissue patterns. Malignant tumors often display complex, heterogeneous tissue structures, resulting in pronounced variations in pixel intensity. Based on these distinctions, this study identified three primary feature categories, as outlined in Table 1.

- Texture Features: A Gray Level Co-occurrence Matrix (GLCM) was employed in four directional angles (0°, 45°, 90°, and 135°) to extract a set of 22 statistical features. These features encapsulate detailed spatial and statistical distributions of pixel intensities in neighboring regions, serving as key indicators of tissue characteristics.
- Histogram-Based Features: These attributes quantify the distribution of pixel intensities across the image, capturing essential aspects of brightness, contrast, and intensity variation, which contribute to image complexity.
- Shape Features: These features describe the geometric properties of the tumor.

### 3.3. Feature Selection by ISA

In Feature selection, a key step in designing pattern recognition systems, is employed to reduce data dimensionality and enhance classification accuracy. This process involves identifying an optimal subset of features from an initial set, ensuring maximal retention of class-related information while minimizing redundancy and overlap. Feature selection methods are broadly categorized into filter-based and wrapper-based approaches. In filter-based techniques, features are evaluated independently of the classification algorithm based on criteria such as correlation, interdependence, and statistical consistency, selecting the most discriminative attributes. Representative methods include Fast Correlation-Based Filters (FCBF), Correlation Feature Selection (CFS), and consistency-based filters [14]. These techniques are computationally efficient and particularly suited for high-dimensional datasets. Conversely, wrapper-based methods involve direct interaction with classifier performance, where the quality of each feature subset is assessed based on classification accuracy. Although these methods generally yield superior feature selection results compared to filter-based approaches, their high computational cost limits their widespread application [15].

In recent years, the use of metaheuristic algorithms for feature selection has expanded significantly, particularly in the analysis of medical data. A high-dimensional feature space can lead to increased class overlap, adversely affecting classification accuracy. To mitigate this issue, metaheuristic algorithms offer an effective approach for identifying an optimal subset of the most discriminative features [8,9,10,11].

This research used the metaheuristic SA algorithm to address the feature selection problem. SA is a probabilistic search algorithm inspired by the gradual cooling process in metallurgy. Its ability to escape local optima by accepting suboptimal solutions during the early search phases makes it well-suited for complex optimization problems, such as feature selection [4,16]. However, the conventional SA framework may struggle with high-dimensional datasets, particularly those involving medical imaging. To enhance its feature selection efficiency, this research introduces two structural modifications to the standard SA algorithm.

- Defining a composite fitness function

In the conventional SA framework, the fitness function is solely determined by classification accuracy. In the proposed approach, the cost function is formulated as a combination of classifier accuracy and the ratio of selected features to the total feature set, as expressed in Equation (1). Consequently, beyond improving classification accuracy, the algorithm prioritizes selecting a more compact subset of features, thereby reducing model complexity and enhancing generalizability.

(1) $$\mathrm{fitness}=\left(1-\mathrm{Acc}\right)+\beta \frac{\mathrm{nf}}{D}$$

where Acc represents classification accuracy, nf is the number of selected features, D denotes the total number of features, and β is a parameter that controls the trade-off between feature reduction and accuracy, ranging from 0 to 1. By employing this cost function, the algorithm aims not only to maximize classification accuracy but also to minimize the size of the final feature set.

- Dynamic and unfixed selection of feature number

In the ISA algorithm, feature selection is executed dynamically and optimally. Unlike the classic SA version, where the number of selected features is fixed and predetermined, ISA determines this number randomly and in a self-regulated manner at each iteration. To accomplish this, a random value, nf—representing the number of selected features—is drawn from a predefined interval (e.g., 10 to 40) at the start of the cost function evaluation. Subsequently, the features are ranked by the algorithm from best to worst, and the top nf features are chosen to constitute the current subset. In addition to computing classification accuracy using this dynamic subset, the ISA cost function includes a penalty term proportional to the ratio of selected features to the total number of features. This formulation guides the algorithm not only to enhance accuracy but also to avoid redundant and unnecessary features. Furthermore, by varying the length of the feature subset in each iteration and reconstructing neighbors—that is, generating new samples in the vicinity of the current solution with a different subset length—the algorithm dynamically analyzes the underlying data structure and inter-feature relationships to identify the optimal feature combination. This mechanism enhances the algorithm’s flexibility, bolsters the model’s generalizability, and mitigates overfitting. The dynamic, self-regulated selection of the feature count is primarily implemented within the cost function’s evaluation routine. At the beginning of each execution, nf is randomly chosen from the predetermined interval, and the feature subset is subsequently formed based on this value. During the neighbor reconstruction phase, the number of selected features is incrementally adjusted (for example, by adding or subtracting 1 or 2), ensuring that nf remains within the defined limits from nf_min to nf_max. These incremental adjustments enable the algorithm to explore the vicinity of the current solution and evaluate different feature subsets, thereby avoiding local optima and discovering superior feature combinations. Consequently, neighbor reconstruction significantly enhances the ISA algorithm’s flexibility and facilitates more optimal performance in selecting both the number and the combination of features.

The pseudocode for the algorithm that dynamically selects the number of features in ISA is presented in Algorithm 1.

**Algorithm 1.** Pseudocode of the ISA algorithm
**Input:**
 F: Set of all features (size = D) nf_min, nf_max: Range for number of selected features max_iter: Maximum number of iterations β: Weights for cost function component
**Initialize:**
 best_subset ← empty set best_cost ← infinity**For** iter = 1 to max_iter do:  1. Randomly select the number of features:  nf ← random integer between nf_min and nf_max  2. Rank the features based on their scores (e.g., importance, relevance):  F_sorted ← sort F based on feature scores in descending order  3. Select subset S:  S ← first nf features from F_sorted  4. Evaluate classifier accuracy using features in S:  accuracy ← calculate classification accuracy with S  5. Compute cost function:  cost ← (1 − accuracy) + β * (nf / D)  6. If cost < best_cost then  best_cost ← cost  best_subset ← S  7. Neighbor reconstruction for improved search:
-Slightly modify nf by adding or subtracting a small value (e.g., ±1 or ±2), ensuring nf stays within [nf_min, nf_max].-Re-select subset based on new nf and repeat steps 3 to 6.-This exploration helps the algorithm to escape local minima and find better feature subsets dynamically.

**End For**

**Output:**
 best_subset

### 3.4. Classification

In the proposed system, SVM is used to classify tumors as either cancerous or non-cancerous. SVM is one of the most widely utilized supervised learning methods for various classification and regression tasks, prized for its high accuracy and robust generalizability [17,18,19,20]. By identifying an optimal hyperplane, the algorithm segregates the data such that the margin between classes is maximized, thereby enhancing model accuracy and performance on new datasets.

When the data cannot be linearly separated, a nonlinear SVM is employed. In these cases, kernel functions map the data into a higher-dimensional space, enabling effective segregation. This research employs the Gaussian Radial Basis Function (RBF) kernel because of the specific data structure, as it performs well in classification tasks. Additionally, the concept of a soft margin is implemented by introducing a penalty parameter (C) to prevent overfitting and enhance generalizability. This approach permits some errors during training, which in turn improves the accuracy of classifying test samples.

## 4. Results

Images from two well-regarded databases, MIAS and CBIS-DDSM, were utilized to implement and evaluate the proposed method. All processing was conducted in MATLAB (R2018b). The MIAS database contains 322 MLO-view mammograms from 161 patients, stored at a resolution of 1024 × 1024 pixels with an 8-bit depth. This dataset includes 208 normal images, 63 images of benign tumors, and 51 images of malignant tumors. For each image, data detailing tumor location, tissue type, and classification (benign or malignant) is provided in an accompanying text file [21]. The CBIS-DDSM database, an updated and standardized version of DDSM, comprises 3161 mammographic images in DICOM format with a 16-bit depth. This dataset includes images of benign and malignant tumors as well as microcalcifications, captured from standard MLO and CC views. Additionally, ROI images are provided separately. Comprehensive lesion-related information, along with details of the imaging view, is available in a reference file [22]. This study utilized MLO-view images from both databases for system training and evaluation, as this view offers broader breast tissue coverage and is crucial for accurately detecting deep-seated lesions.

To implement and evaluate the proposed method, 100 images from the MIAS database were used, comprising 50 benign and 50 malignant tumor images. Forty samples from each class were allocated for classifier training, while the remaining 10 were reserved for performance evaluation. Thus, 80 images served as training data and 20 as test data. Additionally, 300 images from the CBIS-DDSM database were employed, including 150 benign and 150 malignant tumor images. From each class, 120 samples were used for classifier training, and the remaining 30 for performance assessment, resulting in a total of 240 training images and 60 test images. To enhance evaluation reliability and prevent results from depending on random sample selection, a 5-fold cross-validation strategy was applied to partition the datasets into training and test sets.

First, a median filter with a 3 × 3 mask was applied to the ROI, followed by histogram equalization. In the second stage, 104 features, including tissue, shape, and histogram characteristics, were extracted from the images. Feature selection was performed using SA and ISA methods, with parameters fine-tuned through experimental testing. In both approaches, the maximum replication count was set to 40, the number of sub-iterations to 10, the temperature decay rate to 0.9, the initial temperature to 4, the β value in the cost function to 0.01, and the dynamic range for feature selection in ISA to 10–40. After processing the samples through the proposed system, ISA selected 34 features for the MIAS database and 31 features for the CBIS-DDSM database. To ensure a fair comparison of feature selection efficiency, the SA algorithm was constrained to use the same number of features. Finally, tumor classification was performed using a nonlinear SVM with a radial basis function (RBF) kernel and a soft margin. Optimal values for the kernel width and penalty parameter (C) were selected from the set {0.0001, 0.001, 0.01, 0.1, 1, 10, 100} based on the classifier’s best performance.

The performance of the proposed system in detecting breast cancer tumors was evaluated using accuracy, sensitivity, specificity, and F1 score. These metrics were computed based on values obtained from the confusion matrix [23]. The confusion matrix is constructed by comparing the predicted labels generated by the SVM classifier with the actual labels of the samples. Table 2 provides an example of this matrix, while Table 3 outlines its parameters and the relationships among the evaluation indices.

Given that the 5-fold cross-validation method was employed to assess the performance of the proposed system, Table 4 presents the corresponding confusion matrices for each dataset after the system was applied to the MIAS and CBIS-DDSM databases.

The error matrices presented in Table 4 for the MIAS database demonstrate that the ISA feature selection algorithm not only improved overall accuracy but also proved to be a more reliable option for sensitive applications such as cancer detection. Specifically, it significantly reduced FN and increased TP compared to the SA algorithm and the scenario where all features were utilized. Similarly, the confusion matrices for the CBIS-DDSM database indicate that employing the ISA method for feature selection led to a notable increase in classification sensitivity and accuracy while effectively reducing FN—a critical factor in medical diagnostics.

**Figure 2:**
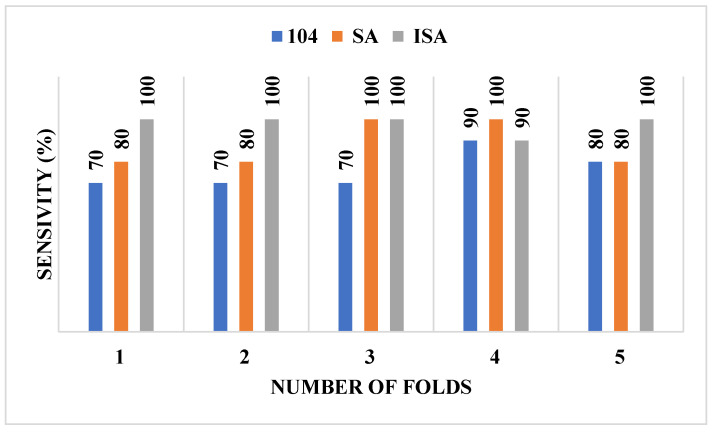
Sensitivity values for each fold for the MIAS dataset.

**Figure 3:**
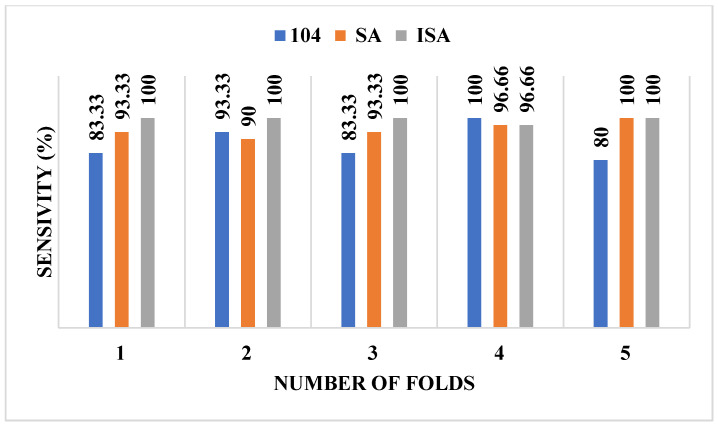
Sensitivity values for each fold for the CBIS-DDSM dataset.

Table 5 presents the mean and standard deviation of assessment indices for feature selection using both ISA and SA methods from an initial set of 104 features. The columns labeled ISA and SA in Table 5 correspond to results obtained using the respective feature selection techniques. The data reveal that, for the MIAS database, mean accuracy, sensitivity, and F1-score were substantially higher when utilizing the 34 features selected via ISA rather than all 104 features or 34 features selected using SA. Likewise, for the CBIS-DDSM database, all evaluation parameters exhibited superior performance when applying 31 features selected via ISA rather than all 104 features or 31 features selected using SA. It is important to note that, for both databases, the SA-based feature selection method achieved significantly better results than using the complete feature set. Figure 2 and Figure 3 illustrate sensitivity trends for the MIAS and CBIS-DDSM databases, respectively, highlighting the superior performance of ISA compared to scenarios where no feature selection is applied.

## 5. Discussion

The proposed system leverages shape, histogram, and tissue-related features, as the shape, margins, and tissue characteristics of masses change in cancerous tumors. Tissue features were extracted in multiple directions to provide more comprehensive information about tumor characteristics. However, certain extracted features were redundant, negatively impacting classification performance and increasing data volume. Therefore, ISA and SA methods were applied to isolate the most relevant features. In the ISA approach, the number of selected features is determined dynamically and adaptively in each iteration, unlike the conventional SA method, where the feature count remains fixed. This adaptive mechanism enables the model to align itself with the structural characteristics of the input data and select an optimal subset of features. Additionally, incorporating classification accuracy with a penalty term tied to the number of selected features within the cost function mitigates model complexity and prevents overfitting. By employing an adaptive search process that balances accuracy and simplicity, ISA effectively selects more relevant and impactful features, directly contributing to higher sensitivity and improved identification of positive cases.

The metaheuristic SA algorithm offers distinct advantages over conventional feature selection techniques, as it occasionally accepts weaker solutions within the search space to avoid trapping in local optima. This paper introduces ISA as an improved version of SA, incorporating a composite cost function to select a feature subset that minimizes feature count while maintaining high classification accuracy. Furthermore, ISA automatically determines the optimal number of features through a dynamic selection mechanism where the feature count is not fixed. Thus, ISA effectively enhances classification performance while reducing data dimensionality.

Sensitivity, a critical parameter in evaluating the performance of medical diagnostic systems, reflects a system’s ability to accurately identify positive cases (patients). Maintaining a high sensitivity level is essential in medical applications, particularly in breast cancer detection, because lower sensitivity results in an increased number of false negatives—cases incorrectly classified as healthy. Such misclassifications can have severe consequences, including delayed diagnosis and the loss of critical time for effective treatment. Therefore, enhancing sensitivity is a fundamental objective in the development of assistive diagnosis systems.

As a result, ISA not only enhances overall classification accuracy but also improves system sensitivity across various conditions, demonstrating greater stability compared to SA or models utilizing all available features.

Table 6 compares the performance of the proposed system with several existing methods. The system achieves more accurate breast cancer tumor detection by extracting diverse features—such as shape, tissue characteristics, and histogram—from suspicious regions in mammograms and employing the ISA algorithm to select the most distinctive features. The integration of ISA for feature selection enhances system efficiency. Unlike the conventional SA method, which maintains a fixed number of selected features, ISA employs a dynamic selection process, allowing the algorithm to adapt to the data structure and determine an optimal feature subset in each iteration. This capability for dynamic feature adjustment not only enhances algorithmic flexibility but also reduces the risk of selecting an excessive number of redundant features, which could otherwise lead to overfitting. Additionally, incorporating a penalty term for the ratio of selected features to total features within the cost function ensures a balanced trade-off between classification accuracy and model simplicity. The performance of the SVM classifier applied to ISA-selected features further underscores the effectiveness of this approach in distinguishing benign tumors from malignant tumors. Building upon these strengths, our method exhibits clear comparative advantages among recent advanced CAD systems evaluated on the MIAS and CBIS-DDSM datasets. The dynamic feature selection of the ISA algorithm prevents redundancy and overfitting by continuously adapting the feature subset, while the penalty term balances accuracy with model complexity. The high classification performance of the SVM with ISA-selected features confirms the robustness of our system. These attributes enable our method to achieve competitive or superior accuracy alongside reduced computational demands, supporting its potential for real-world clinical deployment.

The method presented in [3], similar to our proposed system, employs metaheuristic algorithms for optimizing the classification process, and its accuracy on the INbreast dataset is slightly higher than that of our method. Both methods achieve high accuracy in breast tumor classification, yet they differ in methodology and data usage. The method in [3] employs an ensemble of pre-trained CNN models combined with transfer learning from ImageNet and metaheuristic optimization (GWO and HGS) for hyperparameter tuning. Using the INbreast dataset, this approach achieved an accuracy of 99.9%, benefiting from deep feature representations, data augmentation, and optimized network parameters. Our method, in contrast, employs a classical CAD pipeline that includes ROI extraction, handcrafted shape, histogram, and tissue features, feature selection using the ISA algorithm, and classification with an SVM. Evaluated on the CBIS-DDSM and MIAS datasets, our system achieved accuracies of 99.67% and 98%, respectively. ISA dynamically selects an optimal subset of features, reducing redundancy and overfitting while maintaining high classification performance with lower computational cost. The slightly higher accuracy of the method in [3] can be attributed to the use of deep features and extensive transfer learning. Nevertheless, our method demonstrates advantages in interpretability, efficiency, and practical applicability, as the selected features are directly related to tumor characteristics and the system does not rely on large pre-trained networks. This ensures robustness across diverse datasets and facilitates potential clinical deployment. Both approaches highlight the effectiveness of metaheuristic algorithms in enhancing classification, albeit at different stages: in [3] for hyperparameter optimization and our method for feature selection. Overall, the results obtained from the proposed system demonstrate that it provides a robust strategy for enhancing the performance of machine learning models in high-stakes medical applications.

The developed CAD system is designed for practical clinical application and is fully compatible with widely accepted medical imaging standards, including comprehensive support for DICOM, which facilitates seamless integration with existing radiology systems. To maintain patient confidentiality, automated anonymization processes are applied to data before analysis, ensuring compliance with privacy regulations. The system also supports direct communication with Picture Archiving and Communication Systems, allowing for efficient image handling, secure storage, and easy retrieval of mammograms and related diagnostic information. Technically, the feature extraction and selection modules are designed to retain diagnostically meaningful details within mammograms, including subtle texture and shape characteristics, which are especially critical when analyzing dense breast tissue where abnormalities are harder to discern. By serving as a supplementary reviewer in the radiologist’s workflow, the system can pinpoint suspicious areas, provide quantitative lesion assessments, and reduce variability among observers—thereby bolstering diagnostic accuracy and supporting earlier breast cancer detection. Although current validations utilize publicly available datasets, ongoing work will expand testing to encompass more diverse clinical populations and imaging conditions, enhancing the robustness and clinical readiness of the system.

While demonstrating strong performance, the system has limitations related to dataset diversity and challenges inherent in imaging dense breast tissue. To address these issues, future studies will incorporate more heterogeneous datasets and refine feature extraction and selection methods to better accommodate patient-specific variability. These efforts aim to enhance the system’s applicability and reliability in varied clinical environments, ultimately improving diagnostic outcomes.

## 6. Conclusions

Breast cancer, one of the leading causes of mortality among women worldwide, can be effectively treated if diagnosed at an early stage. This study introduces a CAD system for the automated detection of cancerous tumors in mammograms. The system consists of four stages: preprocessing, feature extraction, feature selection, and classification. In the feature selection stage, SA and ISA algorithms were employed to identify effective and distinguishing features among shape, tissue, and histogram attributes. Evaluation using the MIAS and CBIS-DDSM databases demonstrated that the proposed method achieved an accuracy exceeding 98%, a sensitivity of approximately 99%, and an F1-score close to 98%, indicating strong performance in detecting malignant tumors. These results highlight the system’s high capability in correctly identifying positive cases while minimizing false negatives (FN), a critical factor in sensitive medical applications.

The promising results of this study provide a solid foundation for future research exploring hybrid optimization strategies. The integration of complementary methods can significantly enhance the robustness, generalizability, and adaptability of the feature selection process when dealing with diverse datasets and imaging conditions. Such advancements will be critical for advancing the clinical applicability of the proposed system. In future research, the development and evaluation of hybrid approaches to improve the system’s performance and generalizability will be prioritized.

## Figures and Tables

**Table 1:** Extracted features.

Feature Category	Feature Names	Number of Features
Texture (GLCM)	Autocorrelation, Contrast, Correlation, Correlation: Matlab, Cluster Prominence, Cluster Shade, Dissimilarity, Energy, Entropy, Homogeneity, Maximum probability, Sum of squares: Variance, Sum average, Sum variance, Sum entropy, Difference variance, Difference entropy, Information measure of correlation1, Information measure of correlation2, Inverse difference (INV) is homom, Inverse difference normalized (INN), Inverse difference moment normalized	88
Histogram	Mean, Variance, Skewness, Kurtosis, Energy, Entropy	6
Shape	Solidity, Area, MajorAxisLength, MinorAxisLength, ConvexArea, Eccentricity, Orientation, Perimeter, Extent, EquivDiameter	10

**Table 2:** The confusion matrix.

Category	The Label Predicted by the Classifier	
	Malignant	Benign
Malignant	TP	FN
Benign	FP	TN

**Table 3:** The performance parameters.

Parameters	Concept/Formula
TP	The number of malignant tumors accurately identified as malignant
FN	The number of malignant tumors misclassified as benign
FP	The number of benign tumors misclassified as malignant
TN	The number of benign tumors correctly identified as benign
Accuracy	$Accuracy=\frac{TP+TN}{TP+FP+TN+FN}\times 100$
Sensitivity	$Sensitivity=\frac{TP}{TP+FN}\times 100$
Specificity	$Specificity=\frac{TN}{TN+FP}\times 100$
F1-Score	$F1-Score=2\times \frac{\left(\frac{TP}{TP+FP}\times 100)\times (\;\frac{TP}{TP+FN}\times 100\right)}{\left(\frac{TP}{TP+FP}\times 100)+(\;\frac{TP}{TP+FN}\times 100\;\right)}$

**Table 4:** The obtained confusion matrices.

		Number of Folds									
		1		2		3		4		5	
**MIAS dataset**	**34 selected features using ISA**	10	0	10	0	10	0	9	1	10	0
		0	10	0	10	0	10	0	10	1	9
	**34 selected features using SA**	8	2	8	2	10	0	10	0	8	2
		0	10	0	10	0	10	0	10	0	10
	**104 features**	7	3	7	3	7	3	9	1	8	2
		1	9	0	10	1	9	0	10	1	9
**CBIS-DDSM dataset**	**31 selected features using ISA**	30	0	30	0	30	0	29	1	30	0
		0	30	0	30	0	30	0	30	0	30
	**31 selected features using SA**	28	2	27	3	28	2	29	1	30	0
		1	29	0	30	2	28	0	30	1	29
	**104 features**	25	5	28	2	25	5	30	0	24	6
		5	25	0	30	5	25	5	25	5	25

**Table 5:** Mean and standard deviation for the evaluation parameters obtained from SVM classification.

Dataset		MIAS			CBIS-DDSM		
Feature Selection Method/Number of Features		ISA/34	SA/34	104	ISA/31	SA/31	104
Accuracy (%)	Average	98	94	85	99.67	96	87.33
	Standard deviation	2.74	5.48	6.12	0.75	2.24	6.52
Sensitivity (%)	Average	98	88	76	99.33	94.67	88
	Standard deviation	4.47	10.95	8.94	1.49	3.8	8.37
Specificity (%)	Average	98	100	94	100	97.33	86.67
	Standard deviation	4.47	0	5.4	0	2.79	7.45
F1-score	Average	97.9	93.33	83.86	99.66	95.93	87.15
	Standard deviation	2.88	6.09	6.59	0.76	2.26	6.37

**Table 6:** Comparison of the classification performance of the proposed method with other existing methods.

Author	Utilized Dataset	Feature Selection/Optimizing Hyperparameters	Classifier	Accuracy	Key Advantages
Thirumalaisamy, 2023 [6]	MIAS/CBIS-DDSM	ACO, OBL	CNN	98.63%, 99.15%	Metaheuristic optimization, transfer learning, CNN-based classifier
Dumbare, 2023 [7]	MIAS/DDSM	GOA	CNN	96%	Grasshopper Optimization Algorithm, deep learning model
Alnowaiser, 2024 [8]	MIAS/INbreast	GWO	CNN	99.86%, 99.4%	Advanced optimization, high accuracy with CNN
Bilal, 2024 [9]	MIAS	GWO	SVM	99.25%	Optimization with GWO, traditional SVM classifier
Torabi Jafroudi, 2024 [10]	MIAS	GA-MOO	ANN	98.2%	Multi-objective optimization, ANN classifier
Kanya Kumari, 2024 [11]	MIAS	TLBO	ANN, KNN, RF, SVM, XGBoost	93.9%, 92.7%, 94.8%, 93.8%, 97.8%	Multiple classifiers, varied optimization
Sreevani, 2025 [12]	CBIS-DDSM/BC	AO	GCRNN	99.65%	Hybrid recurrent neural network with AO optimization
Saber, 2025 [3]	INbreast	GWO, HGS	CNN/SVM	99.9%	Metaheuristic-based CNN/SVM hybrid
Proposed method	MIAS/DDSM	ISA	SVM	98%, 99.67%	Dynamic feature selection reducing redundancy, penalty term in cost function, balanced accuracy and simplicity

## Data Availability

The datasets used and analyzed during the current study are publicly available. The MIAS dataset can be downloaded from Kaggle at https://www.kaggle.com/datasets/kmader/mias-mammography (see [21]) (accessed on 28 August 2015), and the CBIS-DDSM dataset is accessible at https://www.cancerimagingarchive.net/collection/cbis-ddsm/ (see [22]) (accessed on 14 September 2017). Both datasets are open for research use.
